# Automation in tibial implant loosening detection using deep-learning segmentation

**DOI:** 10.1007/s11548-025-03459-1

**Published:** 2025-06-27

**Authors:** C. Magg, M. A. ter Wee, G. S. Buijs, A. J. Kievit, M. U. Schafroth, J. G. G. Dobbe, G. J. Streekstra, C. I. Sánchez, L. Blankevoort

**Affiliations:** 1 Quantitative Healthcare Analysis (QurAI) Group, Informatics Institute, University of Amsterdam, Amsterdam, The Netherlands; 2 Biomedical Engineering and Physics, Amsterdam UMC Location University of Amsterdam, Amsterdam, The Netherlands; 3 Department of Orthopedic Surgery and Sports Medicine, Amsterdam UMC Location University of Amsterdam, Amsterdam, The Netherlands; 4 Amsterdam Movement Sciences, Musculoskeletal Health, Amsterdam, The Netherlands

**Keywords:** Total knee arthroplasty, Aseptic loosening, Deep learning, Segmentation

## Abstract

**Purpose:**

Patients with recurrent complaints after total knee arthroplasty may suffer from aseptic implant loosening. Current imaging modalities do not quantify looseness of knee arthroplasty components. A recently developed and validated workflow quantifies the tibial component displacement relative to the bone from CT scans acquired under valgus and varus load. The 3D analysis approach includes segmentation and registration of the tibial component and bone. In the current approach, the semi-automatic segmentation requires user interaction, adding complexity to the analysis. The research question is whether the segmentation step can be fully automated while keeping outcomes indifferent.

**Methods:**

In this study, different deep-learning (DL) models for fully automatic segmentation are proposed and evaluated. For this, we employ three different datasets for model development (20 cadaveric CT pairs and 10 cadaveric CT scans) and evaluation (72 patient CT pairs). Based on the performance on the development dataset, the final model was selected, and its predictions replaced the semi-automatic segmentation in the current approach. Implant displacement was quantified by the rotation about the screw-axis, maximum total point motion, and mean target registration error.

**Results:**

The displacement parameters of the proposed approach showed a statistically significant difference between fixed and loose samples in a cadaver dataset, as well as between asymptomatic and loose samples in a patient dataset, similar to the outcomes of the current approach. The methodological error calculated on a reproducibility dataset showed values that were not statistically significant different between the two approaches. The results of the proposed and current approaches showed excellent reliability for one and three operators on two datasets.

**Conclusion:**

The conclusion is that a full automation in knee implant displacement assessment is feasible by utilizing a DL-based segmentation model while maintaining the capability of distinguishing between fixed and loose implants.

**Supplementary Information:**

The online version contains supplementary material available at 10.1007/s11548-025-03459-1.

## Introduction

Patients with a total knee arthroplasty (TKA) undergo revision surgery in 13% of all cases within 10 years [1]. One of the main reasons is the indication of implant loosening, occurring in approximately 30% of the cases [2, 3]. The diagnostic process of aseptic loosening can include conventional radiography, computer tomography (CT) scan, bone scintigraphy, positron emission tomography with CT (PET-CT), single-photon emission computed tomography with CT (SPECT/CT) and magnetic resonance imaging (MRI) [4]. These imaging modalities merely show indirect signs of loosening and can lead to incorrect diagnosis, which can cause either undertreatment or unnecessary surgeries [4]. Up until recently, there was no method to measure loosening in a clinical diagnostic setting. Only the migration of an implant over time could be measured. The methods to quantify implant migration are marker-based Roentgen stereo photogrammetric analysis (RSA) and model-based RSA [5]. Currently, model-based RSA is the standard involving the placement of radiopaque markers into the bone of interest at the time of TKA surgery. These markers act as well-defined artificial landmarks, allowing for the tracking of the bone’s position and orientation relative to the 3D model of the implant, which provides information about migration of the implant over time. Due to the invasive nature, this method is solely used in research settings and is not suitable for population-wide or diagnostic use. The Implant Movement Analysis (IMA, Sectra Inc., Linköping) [6] uses CT scans of the knee forced to internal and external rotation, and to varus and valgus position, to analyse implant displacement. The leg is held in position by straps and cushions or manually by an examiner. A semi-automatic tool performs a landmark-based rigid body transformation between two scans, which is then visually examined for prosthesis movement. Parameters to quantify the displacements were not reported.

A non-invasive method to directly measure and quantify the tibial implant displacement was recently introduced by Kievit et al. [7] and further evaluated by Buijs et al. [8]. Two CT scans of the knee are acquired under varus and valgus loading using a loading device. The scans are subsequently analysed by an advanced 3D image analysis workflow, which consists of segmentation and registration to quantify implant displacement relative to the tibia between varus and valgus loading. Especially the semi-automatic segmentation step requires user interaction, such as manual corrections of segmentation errors due to metal artefacts in the CT scans. This task can become tedious and time-consuming when the image is significantly hampered by metal artefacts.

In recent years, machine learning and deep learning (DL) have already been used for various tasks related to knee arthroplasty [9]. Among others, DL-based detection of implant loosening on X-Rays have been introduced [10–12]. However, due to their 2D nature, they only estimate a loose or fixed classification without quantifying load-induced displacement which provides additional information to a surgeon.

The aim of this study was to contribute to the image analysis workflow as described by Buijs et al. [8] by adding an increased level of automation by replacing the current semi-automatic segmentation approach with a fully automatic segmentation method. The question was whether the displacement analysis workflow with a fully automatic segmentation model results in outcomes that were not different from the outcomes of the current workflow using semi-automatic segmentations. The approach was to train and evaluate 2D and 3D segmentation models with three different segmentation annotation protocols to establish the best segmentation protocol considering not only the DL-task of semantic segmentation but also the downstream task of image registration and displacement quantification. An evaluation on patient data was performed to test the performance on real-world data.

## Method

**Fig. 1:**
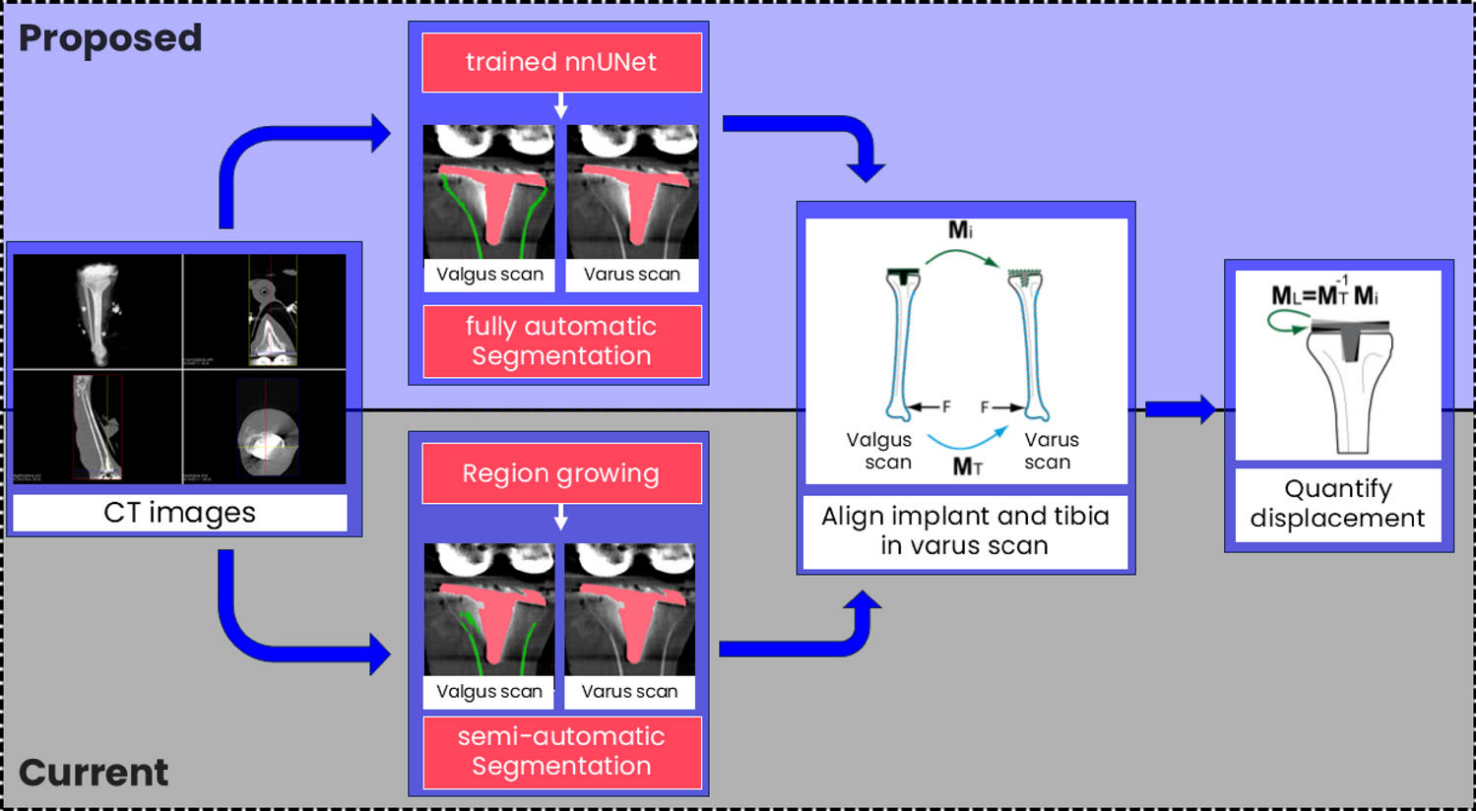
Overview of the current and the proposed displacement workflow: In the current workflow [8] the segmentation masks are generated by a semi-automatic algorithm. For the proposed workflow, a fully automatic DL segmentation model (trained nnUNet) generates the required segmentation masks

**Fig. 2:**
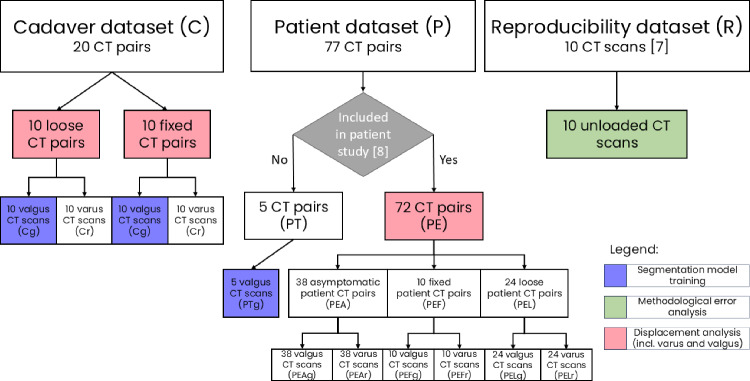
Overview of the dataset composition: cadaver dataset (*C*), patient dataset (*P*) and reproducibility dataset (*R*). The blue, green, and red boxes highlight the subsets that are used for segmentation model training, methodological error analysis, and displacement analysis, respectively

This study was an extension of the work by Kievit et al. [7] and Buijs et al. [8], which describes a current workflow to quantify the load-induced displacement of the tibial implant based on two CT scans. The segmentation masks generated by a semi-automatic approach were replaced in the proposed approach with predictions of a DL-model, i.e. trained nnUNet (Fig. 1). Three different datasets were used for model development and evaluation, i.e. a cadaver dataset (*C*), a patient dataset (*P*) and a reproducibility dataset (*R*) (Fig. 2). Reference labels for the tibia bone and implant were generated semi-automatically for the training dataset of the segmentation model. In the following subsections, details about the dataset acquisition & preparation, data annotation & processing, current approach, segmentation model, proposed approach and performance assessment are discussed.

### Datasets acquisition & preparation

The cadaver dataset (*C*) was acquired in an earlier study [7]. Ten thawed, previously fresh-frozen cadaver legs were equipped with a total knee prosthesis (Vanguard, Zimmer Biomet, Warsaw, Indiana, United States). To simulate a loose implant, the TKA was first loosely implanted without bone cement and scanned under loading. A loaded CT scan was acquired by strapping the knee into a loading device. Then, a moment of force (20 Nm) was applied in valgus and varus direction which induces a displacement of the tibial component relative to the tibia bone. One CT scan was acquired for each load direction, providing a pair of CT scans for each analysis. Then, the TKA was removed and fixated with bone cement. The imaging protocol with valgus and varus loading was repeated. Therefore, in total 20 CT scan pairs were acquired, i.e. 20 CT scans under valgus (Cg) and 20 under varus (Cr) loading belonging to 10 cadaveric specimens.

The patient dataset (*P*) contained 77 CT pairs obtained in a single-centre clinical study at the Amsterdam UMC [8] following the same imaging and loading protocol as for the cadaver study [7]. This set contains various brands and models of TKAs. 38 CT pairs were from asymptomatic patients (PEA, i.e. patient evaluation asymptomatic), which did not report complaints during their last outpatient visit and therefore did not undergo revision surgery. Thus, it is unknown whether the implant was loose or fixed. 34 CT pairs were from symptomatic patients suspected for aseptic loosening (PEL + PEF, i.e. patient evaluation dataset loose and fixed). During their revision surgery, the orthopaedic surgeon evaluated whether the implant was loose (PEL) or fixed (PEF). This intraoperative finding was available as a reference for loosening. 5 CT pairs were inappropriate for the displacement analysis due to technical scan issues or patients not receiving revision surgery and therefore excluded from the initial clinical study [8], but included in the training dataset (PT) (see Section Data Annotation & Processing). All patients gave informed consent for the reuse of their data for related research and Medisch Ethische Toetsingscommissie (METC, Medical Research Ethics Committee) approval (2014_279) for this study was acquired.

The reproducibility dataset (*R*) contained 10 CT scans from one frozen cadaver specimen with a knee prosthesis [7]. The cadaver leg was scanned 10 times without loading but slightly displaced on the CT table between scans. Therefore, any detected displacement of the implant with respect to the bone, was due to methodological error.

The CT scans were acquired with a Philips Brilliance 64-channel CT Scanner (Philips Healthcare, Best, The Netherlands) or a Siemens SOMATOM Force (Siemens Healthineers, Forchheim, Germany). A standard CT scanning protocol of the knee was used, using 160 mAs, 120 kV, balancing image quality and dose. For reconstruction, a relatively sharp bone kernel (D for Philips and Br 64 for Siemens) was used. The isotropic voxel spacing was 0.45 mm.

### Data annotation & processing

**Fig. 3:**
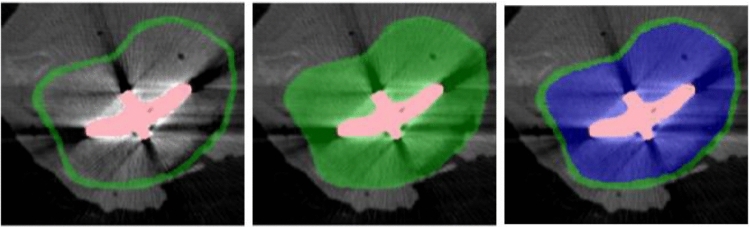
Overview of different tibia segmentation masks: left—cortical tibia bone (Cortical); middle—full tibia bone (Full); right—combination of cortical bone and cortical bone subtracted from full bone (Multi-Class). The implant is displayed in red, the cortical/full tibia bone in green, the combined cortex and inside in green and blue, respectively

A single annotator with five years of experience in healthcare AI and segmentation annotation generated segmentation masks of the tibial implant and tibial bone were generated as reference standard for the training of the segmentation model. The training dataset consisted of 25 valgus loaded CT scans, which includes the 20 valgus loaded CT scans of the cadaver dataset (Cg) and, for increased dataset variability, the valgus loaded CT scans of 5 patient CT pairs (PTg), which were excluded from the patient study [8]. Varus scans closely resembled valgus scans and add little diversity to training, so they were excluded to reduce the annotation effort. The annotation protocols were defined in advance: First, an in-house 3D annotation software using a threshold-based region growing algorithm was employed to accelerate the annotation process by producing intermediate masks [13]. Subsequently, the intermediate masks were manually corrected and refined using ITK-Snap (version 3.8.0, PICSL (University of Pennsylvania)) [14]. There were three alternative tibia segmentation masks (Fig. 3), which built upon each other: The first takes only the cortical tibia bone to mimic the semi-automatic segmentation (Cortex). The seconds fills the cortical bone to create a full tibia bone mask excluding the implant component (Full) with the assumption that it is an easier DL task to segment an entire object. The third divides the full mask into cortical bone and trabecular bone (Multi-Class) to create a multi-class DL-task for bone segmentation. The implant was excluded from the bone segmentation.

The displacement analysis was performed by trained operators. For the cadaver and the reproducibility dataset (C + R), the analysis was performed by one operator (CM) due to time constraints. The remaining 72 patient CT pairs (PE) were analysed by three operators, namely AW, GB and CM (denoted as O1, O2, O3 in no specific order). The analysis was performed separately and independent of each other but following the same analysis protocol.

### Current approach

The current non-invasive direct method [7, 8] to quantify and visualize relative displacement of the tibial implant processes a pair of valgus and varus loaded CT scans with an advanced 3D image analysis workflow. First, the segmentation masks of the tibial cortex and the tibial implant in the valgus scan and the tibial implant in the varus scan are generated with threshold-based region-growing algorithm. The user is required to set a seed point and can adjust the threshold for the semi-automatic segmentation algorithm. In case of strong metal artefacts in the CT scan that influence the segmentation, the user needs to make manual corrections to avoid interference with the registration. Subsequently, a polygon mesh is created from the surface of the segmentation masks by the Marching Cubes algorithm [15]. Using the implant mesh from the valgus scan, the contours of the valgus scan are roughly aligned onto the varus scan with Iterative Closest Point registration [23]. In case of failure, this automatic step can be performed manually by the user. After rough alignment, a double-contour is sampled from the grey-level valgus scans with a + 0.3 mm and − 0.3 mm offset of the polygon surface. Then, point-to-image registration is performed, in which the grey-level values of the double-contour points of the tibial and the implant mesh from the valgus scan are registered to the varus scan. For this, the Nelder-Mead downhill simplex optimization algorithm [16] with a six-parameter search space is used. The optimization is stopped either when a predefined tolerance in correlation coefficient changes is reached or after a set maximum number of iterations.

To quantify the motion of the implant relative to the tibial bone, values of three clinically used displacement parameters [8] are calculated and reported. Registration of a segmentation object (i.e. implant, tibia) from the valgus scan to the varus scan provides a mathematical 4 × 4 repositioning matrix, which represents a rigid transformation as a combination of rotation and translation in 3D. This matrix can be used to calculate the screw axis, an imaginary axis in 3D space, where the object movement is described by a simultaneous translation and rotation, and the magnitude of the rotation about the screw axis (rScrew) [17]. The maximum total point motion (MTPM) describes the largest displacement within the set of points on the implant polygon mesh. The mean target registration error (mTRE) is the average distance of all points on the implant polygon mesh between valgus and varus scan. In addition, a 3D model is created from the polygon mesh and color-coded with a heatmap representing local displacement values from 0 mm (blue) to 0.5 mm and higher (red) (Online Resource, Fig. 6).

### Segmentation model

To automatically generate segmentation masks of the tibial bone and the tibial implant, DL-based segmentation models were used in our study. The base segmentation model was the widely used nnU-Net [18], which is a framework combining a UNet implementation, fixed parameter settings and a data fingerprint extraction to determine rule-based hyperparameters. In total, six different models were trained, i.e. the configurations 2D and 3D full resolution with three different sets for the reference labels, i.e. Cortical, Full and Multi-Class, resulting in the models Cortical 2D, Cortical 3D, Full 2D, Full 3D, Multi-Class 2D, and Multi-Class 3D. More details about the training settings can be found in the Online Resource, Section B. The raw predictions were postprocessed by only keeping the largest connected component per class. The segmentation models were trained on 25 valgus loaded CT scans (Cg + PTg) in a fourfold patient-based cross-validation data split, i.e. the data were split randomly into four parts, using each once for testing and the rest for training (Online Resource, Fig. 7). Thus, for each of the six different configurations, four nnU-Net versions were provided as a first step, trained with the same settings on different partitions of the training dataset. For testing on new data, e.g. the patient evaluation (PE) or the reproducibility (*R*) dataset, the models were combined to an ensemble, i.e. the predictions of the four model versions were aggregated by union to one final segmentation mask. After the training process was finished, the ensemble and the individual nnU-Net versions were referred to as “trained nnU-Net models”.

### Proposed approach

In the proposed approach, the semi-automatic segmentation step of the current approach was now replaced by the predictions of a trained nnU-Net model (Fig. 1). After the prediction by the nnU-Net model, as described previously, the following postprocessing steps are performed with python (3.9.16): First, all but the largest component were removed. Second, to avoid overlap between the bone and the implant mask, a margin was created at the bone implant boundary. To this end, the implant mask was dilated with a circular structural element with a radius of at least 3 mm. Finally, the bone mask overlapping with this area was removed. The resulting segmentation mask was turned into a polygon mesh by the Marching cubes algorithm [15]. The subsequent registration and calculation steps remained the same as previously described.

### Performance assessment

The segmentation performance of the models was assessed against the reference labels with Dice similarity coefficient (DSC) and 95%-percentile Hausdorff Distance (HD95). They were calculated on the fourfold cross-validation scheme in the annotated dataset (Cg + PTg).

For the cadaver dataset (*C*), it was known whether the implant is loose or fixed. The goal of both approaches, i.e. current and proposed, was to separate those two groups. For both groups, the distribution of the displacement parameters were visualized by box plots, the median and the 95% confidence interval (CI) estimated using bootstrap (R = 1000) were reported. The Wilcoxon signed-rank test for pairwise comparison between fixed and loose samples was applied for both approaches. Due to multiple comparisons, the Bonferroni correction of the α-value was applied, i.e. α-value was 0.05/6 = 0.008. Intra-class correlation (ICC) values (two-way mixed effects, absolute agreement, single measurement) [19] were calculated between the parameters of the current and proposed approach. Excellent reliability was indicated by values > 0.90.

The methodological error of displacement parameters of a method was determined on the reproducibility dataset (*R*). Each one of the 10 unloaded scans was registered to its subsequent scan. Due to the absence of displacement, the methodological error was defined by the measured apparent displacement between two sets of CT scans, which theoretically should be 0. For both approaches, the distribution of the error values was visualized by box plots, the median with bootstrapped 95% CI were reported. The pairwise comparison between results of the current and proposed approach was analysed by the Wilcoxon signed-rank test with an α-value of 0.05.

The final model was evaluated on the patient evaluation dataset (PE). Both approaches should distinguish between the subgroups symptomatic loose and asymptomatic (L vs. A) and symptomatic loose and symptomatic fixed (L vs F). The Mann–Whitney U test was used with the Bonferroni corrected α-value of 0.05/24 = 0.002. The pairwise differences between all 4 operators were analysed with the Wilcoxon signed-rank test with a Bonferroni corrected α-value of 0.05/18 = 0.0027. ICC values (two-way mixed effects, absolute agreement, single measurement) were calculated between all four operators.

Statistical significance was indicated in plots with “ns” for *p* values >  = 0.05, * for *p* values < 0.05 and ** for *p* values < α-value (Bonferroni corrected critical value). The statistical analysis was performed in python 3.9.16 with the libraries pyirr and scipy.

## Results

Considering the segmentation performance and the results of the displacement analysis on the cadaver and reproducibility dataset (*C* + *R*), the model Cortex 3D was selected as the final model (see Online Resource, Sect. C). Unless specified otherwise, the results of the proposed approach were acquired using the final model Cortex 3D for automatic segmentation.

### Segmentation

All six segmentation models achieved an overall performance of at least 94% DSC and a maximum of 0.95 mm HD95, with the final segmentation model (Cortex 3D) reaching 95.56% DSC and 0.58 mm HD95 (Table 1). The good segmentation performance of the predictions was also reflected visually in qualitative examples (Online Resource, Fig. 9).

**Table 1:** DSC and HD95 of all six segmentation models for the averaged foreground labels and the individual classes, i.e. tibial cortex and tibial implant. Value in brackets correspond to inside part of the multi-class segmentation and was not considered in the average as it was ignored for further processing

Model	DSC (%) ↑			HD95 (mm) ↓		
	Average	Implant	Tibia	Average	Implant	Tibia
Cortex 2D	94.50	95.22	93.87	0.66	0.62	0.71
**Cortex 3D (Final)**	**95.56**	**95.81**	**95.31**	**0.58**	**0.57**	**0.59**
Full 2D	96.97	95.20	98.74	0.81	0.61	1.01
Full 3D	97.47	95.79	99.14	0.55	0.57	0.52
Multi-Class 2D	94.53	95.14	93.91 (97.15)	0.67	0.63	0.70 (1.16)
Multi-Class 3D	95.53	95.75	95.30 (97.83)	0.58	0.58	0.58 (0.81)

The final model (Cortex 3D) is highlighted in bold

### Displacement measurements

The proposed workflow for calculating displacement parameters was compared to the current approach on different datasets, i.e. cadaveric dataset (*C*), reproducibility dataset (*R*), and patient evaluation dataset (PE), to show cross-validation results, methodological error analysis and real-world patient data results, respectively.

### Cadaveric data (*C*) results

**Fig. 4:**
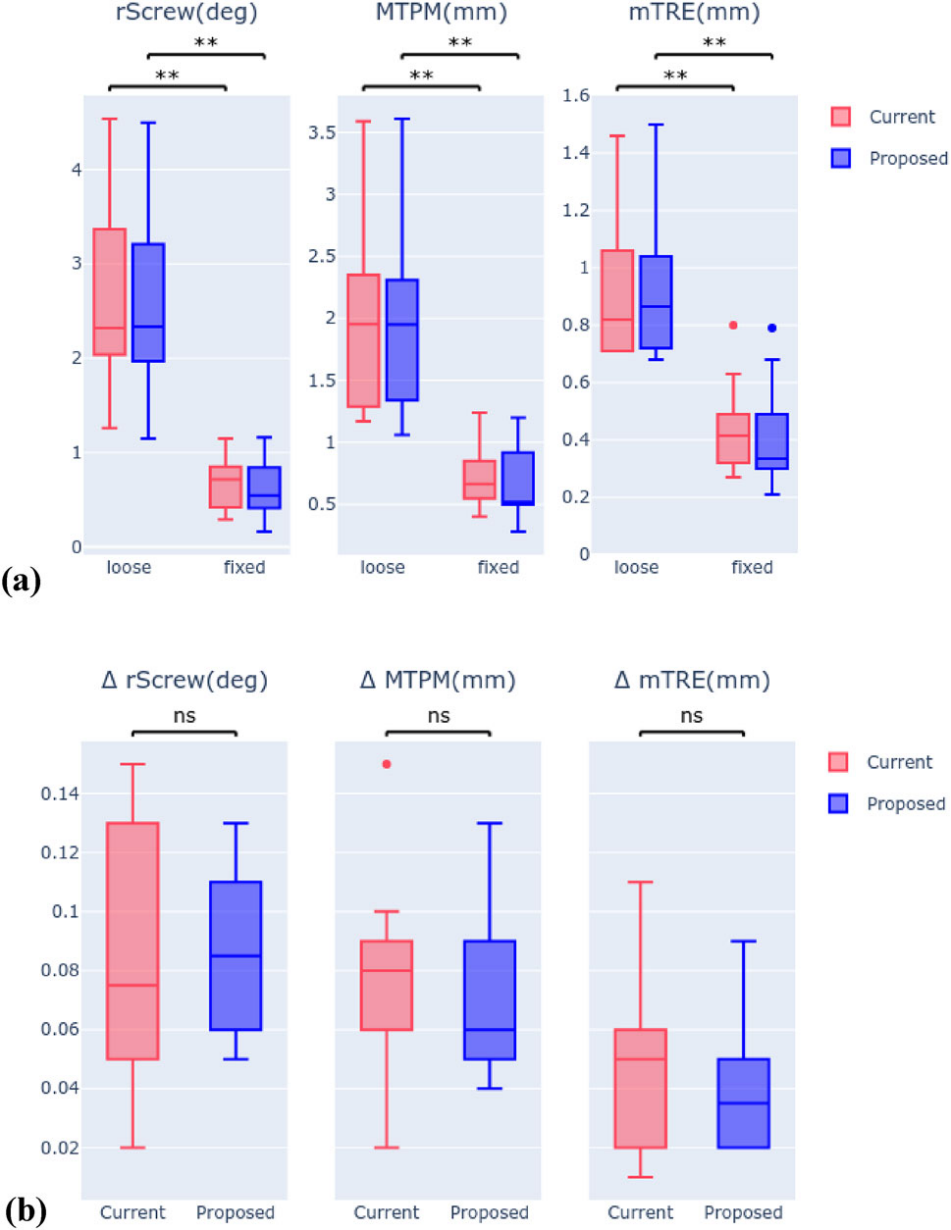
**a** Displacement outcome parameters for loose and fixed cadaveric samples (*C*) and **b** methodological error (∆) for current and proposed workflow, for all three displacement outcome parameters

There was a statistically significant difference between loose and fixed (cemented) samples of the cadaver dataset (C) for both approaches (Fig. 4a, Table 2). The ICC values of the current and proposed approach and the 95% CI were 0.99 [0.975, 0.997] for rScrew(mm), 0.99 [0.979, 0.997] for MTPM(mm), and 0.99 [0.965, 0.994] for mTRE(mm), which indicates excellent reliability for all three displacement parameters.

**Table 2:** Median and 95% CI of displacement outcome parameters for loose and fixed cadaveric samples (C) and *p* values of comparison, for all three displacement outcome parameters ((**) stands for p values < α-value)

Method and measurement	rScrew(deg)	MTPM(mm)	mTRE(mm)
Proposed loose	2.34(1.98–3.20)	1.95(1.57–2.52)	0.87(0.80–1.14)
Proposed fixed	0.55(0.43–0.82)	0.52(0.49–0.82)	0.34(0.31–0.53)
P values Proposed loose vs fixed	0.002(**)	0.002(**)	0.004(**)
Manual loose	2.32(2.04–3.27)	1.96(1.60–2.52)	0.82(0.80–1.11)
Manual fixed	0.72(0.54–0.88)	0.67(0.58–0.87)	0.42(0.36–0.55)
*p* values Manual loose vs fixed	0.002(**)	0.002(**)	0.004(**)

### Reproducibility data (*R*) results

The median and 95% CI of the methodological errors of the proposed approach with the final model Cortex 3D were 0.085° (0.068–0.101) for rScrew, 0.060 mm (0.055–0.088) for MTPM, and 0.035 mm (0.028–0.053) for mTRE, which was not statistically different (p values, respectively: 1.0, 0.31, 0.07) from the current approach, with 0.075° (0.058–0.111), 0.080 mm (0.057–0.098), and 0.050 mm (0.030–0.064), respectively (Fig. 4b; Online Resource, Table 3).

### Patient data (*P*) results

**Fig. 5:**
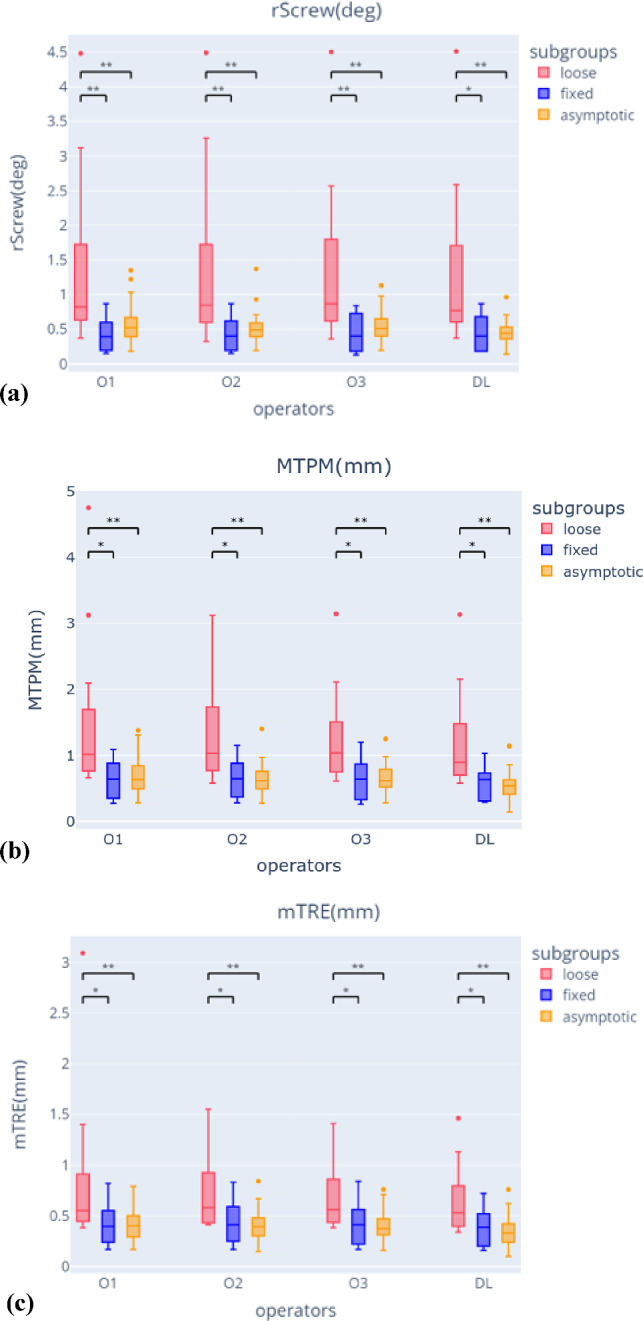
Distribution for the three displacement parameters (**a** rScrew, **b** MTPM, **c** mTRE) grouped by asymptomatic, loose, and fixed patients from the patient evaluation dataset (PE), for the four operators, i.e. three human operators (O1, O2, O3) and DL model

There was a statistically significant difference between loose and asymptomatic samples of the patient dataset (*P*) for all three displacement parameters (i.e. rScrew, MTPM, mTRE) for all four operators (Fig. 5; Online Resource, Table 4 and 5). For the comparison between loose and fixed samples of the patient dataset (*P*), the *p* values were larger than the Bonferroni corrected α value of 0.002 (Fig. 5; Online Resource, Table 4 and 5). The proposed approach underestimated the displacement parameters compared to the current approach executed by three human operators (*p* value < 0.0027; Online Resource, Table 6, Fig. 10). The ICC-values of all four operators and the corresponding 95% CI were 0.99 [0.984, 0.992] for rScrew(deg), 0.96 [0.937, 0.971] for MTPM(mm), and 0.92 [0.892, 0.950] for mTRE(mm), indicating excellent reliability for all measurements.

## Discussion

The primary finding of this study is that the fully automatic DL-based segmentation shows potential to replace the semi-automatic segmentation in the displacement quantification of all patient samples included in this study. DL models have been already used for related segmentation tasks [20–22]. For implant loosening detection, the existing DL-based algorithms use X-ray for classification [10–12]. To the best of our knowledge, this study is the first one to segment TKA components and bone in CT scans for the downstream task of displacement quantification. The displacement parameters (rScrew, MTPM and mTRE) observed in a patient dataset show statistically significant differences between the subgroups (loose, fixed, asymptotic) for both approaches. There is no statistically significant difference between the methodological error of the proposed approach and the current approach (*p* > 0.05). The results of the proposed workflow show excellent reliability with the results of the current approach of one operator on the cadaver dataset and with three operators on the patient evaluation dataset with ICC-values above 0.92.

Comparing different segmentation model settings and segmentation annotation protocols showed three consistent findings in the results (Table 1). First, all 3D models outperform their 2D counterpart with respect to both segmentation metrics. Second, multi-class training did not perform better than single-class training (95.53% DSC for Multi-Class 3D is lower than 95.56% DSC for Cortex 3D and 97.47% DSC for Full 3D). Third, training with full tibia bone masks resulted in better segmentation metrics than tibial cortex bone (97.47% DSC, 0.55 mm HD95 for Full 3D vs.95.56% DSC, 0.58 mm HD95 for Cortex 3D). However, the superior segmentation performance of the Full models did not transfer directly to the downstream task of displacement quantification, as there were two registration failure cases (Online Resources, Fig. 8). Considering all performance metrics, the two best models were Cortex 3D and Full 3D. The final model selection (Cortex 3D) was based on the similarity to the current approach.

The proposed approach underestimated the displacement parameters compared to the current approach (Online Resources Fig. 10). However, in the analysis of the displacement measurement, the exact values of the parameters are less critical since the exact real-world values are unknown. Given the inherently small displacements being measured, there will always be some variability between different operators, whether human or DL-based. Thus, for diagnostic purposes, it is more crucial that the results of the proposed approach are effectively distinguishable between different groups. In this regard, the proposed approach demonstrates the same capabilities as the current approach. As the current method provides displacement quantification, it should not be seen as a tool for implant loosening classification. In order to translate the measurements to classifications and/or probabilities, further work is required. Aside from (multi-variate) threshold analysis [8], DL methods which utilize image and patient data could be explored for this task in the future.

Two limitations of this study are (1) the small number of data samples and (2) the limited variations of implant types in the training set. The training dataset of 25 samples, primarily from a cadaveric dataset, may differ in characteristics from patient data. To demonstrate the applicability of our proposed approach for displacement quantification for patient data, we conducted an extensive evaluation on a patient dataset with broader range of implant types. Despite the limited training set, the proposed approach generalized well. Although we did not evaluate the segmentation mask, visually the model generated predictions only showed smaller geometrical mistakes (Online Resource, Fig. 6 + 8). Nevertheless, further research on the model and proposed approach generalizability are necessary for clinical use.

In the end, the results demonstrate the potential of quantifying load-induced tibial TKA component displacement from CT scans that includes a fully automatic DL-based segmentation to aid the diagnosis of implant loosening. This is the next step towards an automatic workflow for the diagnosis of aseptic knee implant loosening.

## Conclusion

The fully automatic DL-based segmentation shows potential to replace the semi-automatic segmentation of the tibial bone and tibial component of a TKA, with the purpose to quantify load-induced displacement of the tibial TKA component relative to the bone.

## Supplementary Information

Below is the link to the electronic supplementary material.

Supplementary file1 (DOCX 2135 KB)

## Declarations

Authors L. Blankevoort, A.J. Kievit and M.U. Schafroth are listed as inventors of a patent, “Device and Method for Determination of the Moment-induced Movement of a Joint Implant”. Authors L. Blankevoort, A.J. Kievit and M.U. Schafroth own a minority stock of AtMoves BV, an Amsterdam University Medical Center spinoff company.
