# Supplementary material for: Automation in tibial implant loosening detection using deep-learning segmentation
Source: Int J Comput Assist Radiol Surg. 2025 Jun 27;20(10):2065–73. doi: 10.1007/s11548-025-03459-1 (PMC12518389; doi:10.1007/s11548-025-03459-1)
Supplement: Supplementary file 1 — Supplementary file1 (DOCX 2135 KB) [file 11548_2025_3459_MOESM1_ESM.docx]

# Supplementary Material

For “Automation in tibial implant loosening detection using deep-learning segmentation”

C. Magg^1,2,4^, M.A. ter Wee^2,4^, G.S. Buijs^3,4^ , A.J. Kievit^3,4^ , M.U. Schafroth^3,4^ , J.G.G. Dobbe^2,4^ ,G.J. Streekstra^2,4^, C.I. Sánchez^1,2^, L. Blankevoort^3,4^

^1^ University of Amsterdam, Quantitative Healthcare Analysis (QurAI) Group, Informatics Institute, Amsterdam, The Netherlands

^2^Amsterdam UMC location University of Amsterdam, Biomedical Engineering and Physics, Amsterdam, The Netherlands

^3^Amsterdam UMC location University of Amsterdam, Department of Orthopedic Surgery and Sports Medicine, Amsterdam, The Netherlands

^4^ Amsterdam Movement Sciences, Musculoskeletal Health, Amsterdam, The Netherlands

Corresponding Author: Caroline Magg, c.magg@amsterdamumc.nl

# A. Examples

The resulting 3D models with a blue-to-red heatmap encoding the displacement measurement visualize the differences between the current and proposed approach in all three datasets, i.e., a loose and fixed cadaveric sample (Fig. 6a), a sample of the reproducibility dataset (Fig. 6b), two symptomatic and asymptomatic real-world patient scans (Fig. 6c+d).

# B. Training details

**Model architecture** For the purpose of developing an automatic segmentation algorithm, nnU-Net [18] was employed. nnU-Net is a widely used framework, which combines a UNet [24] implementation, fixed parameter settings and a data fingerprint extraction to determine rule-based hyperparameters. It is implemented in PyTorch [25] and handles data preprocessing, model configuration, model training and evaluation. nnU-Net provides several model architecture options; we selected the standard 2D and 3D full-resolution UNet due to their widespread use. Each of the configurations was trained for each of the three different sets of reference labels, i.e., Cortical, Full and Multi-Class (Fig. 3). Thus, in total, six different nnU-Net model configurations were trained, i.e., Cortical 2D, Cortical 3D, Full 2D, Full 3D, Multi-Class 2D, and Multi-Class 3D. Each instance of nnU-Net is referred to as “model” or “DL-model”.

**Model training** The model training was conducted using the nnU-Net framework [18]. For all models, the default data augmentation (i.e., rotations, scaling, Gaussian noise, Gaussian blur, brightness, contrast simulation of low resolution, gamma correction, and mirroring) had be changed to exclude mirroring around the horizontal axes since this was deemed unlikely for knee CT scans. The remaining data augmentation settings have been retained. Stochastic gradient descent with Nestrov momentum (µ=0.99) was used to optimize the network weights with a poly learning rate policy and initial rate of 0.1 over 1000 epochs [26]. In one epoch, 250 mini-batches were sampled from the dataset with foreground oversampling. The loss function was the sum of dice and cross-entropy loss [27].

**Dataset** The training dataset contained 25 valgus loaded CT scans (Section “Data Annotation & Processing”). A 4-fold patient-based cross-validation scheme (Fig. 7) was used, i.e., dataset was split randomly into four equal parts (folds) with the constraint that all samples of a patient were included in one part. Three folds were used for training and the remaining one was used for validation. This process was repeated four times, each time with a different fold used for validation. In the end, for each of the six nnU-Net configurations (i.e., Cortical 2D, Cortical 3D, Full 2D, Full 3D, Multi-Class 2D, and Multi-Class 3D), there existed four versions trained with the same training settings on different partitions of the training dataset.

**Inference** For model inference, i.e., using the trained models to make predictions from new data, the four versions of each nnU-Net configuration were combined to an ensemble, i.e., the predictions of the four versions were aggregated by union to one final segmentation mask. In the case of cross-validation testing, where one fold was reserved for evaluation, instead of the ensemble, only the model version that did not use the evaluation fold during training was used for prediction. This did not only apply for the prediction of the valgus CT scan, but also the corresponding varus CT scan of CT pair. Depending on the context – testing on new data (e.g., reproducibility (R) or patient dataset (P)) or performing cross-validation on the cadaver dataset (C) – the term “trained nnU-Net” refers either to the model ensemble or to the individual model version used in that setting, respectively.

**Implementation Details** The nnU-Net framework was installed with python 3.9.16 and PyTorch 2.0.0**.** The training and validation was performed on an NVIDIA Geforce RTX 2080 Ti 12GB and an Intel Core Xeon Gold 6128 3.40GHz CPU.


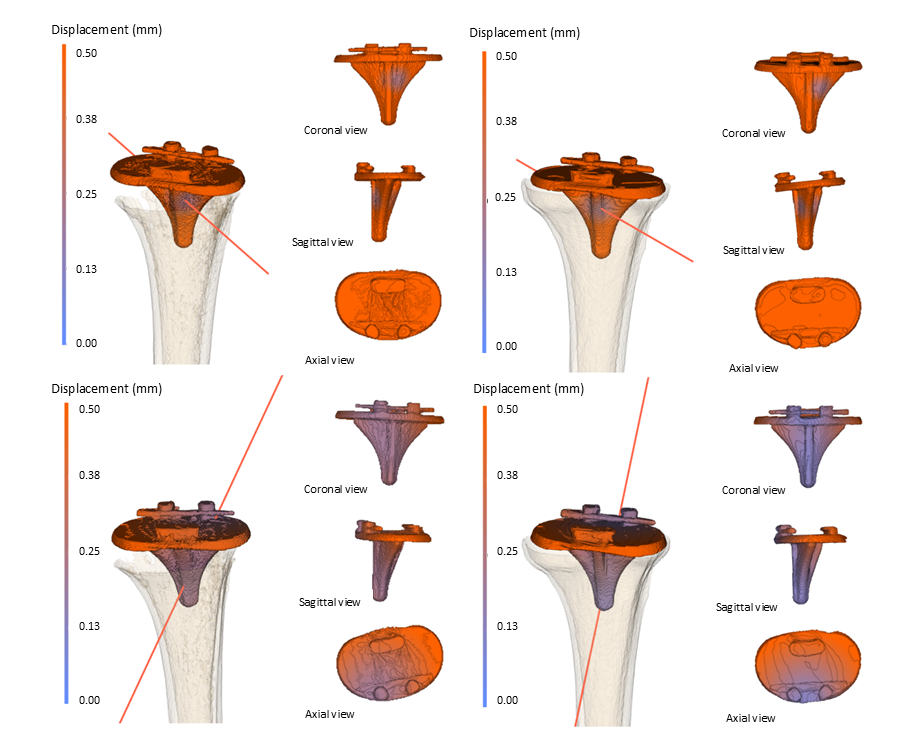
(a) Loose (first row) and fixed (second row) sample of the cadaveric dataset (C).


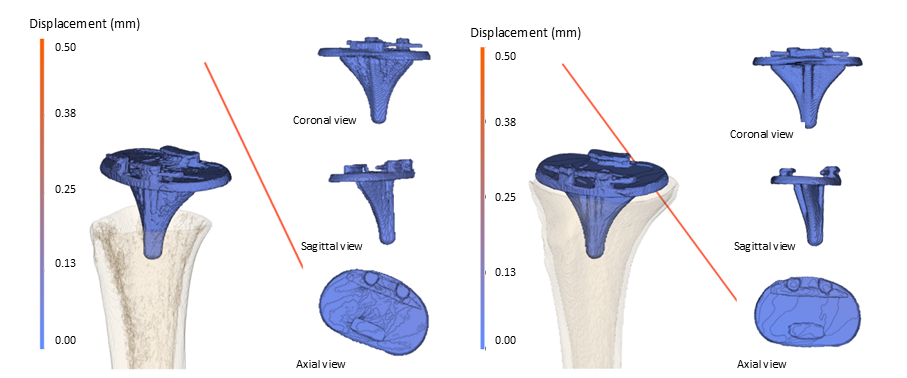
 (b) Sample of the reproducibility dataset (R).


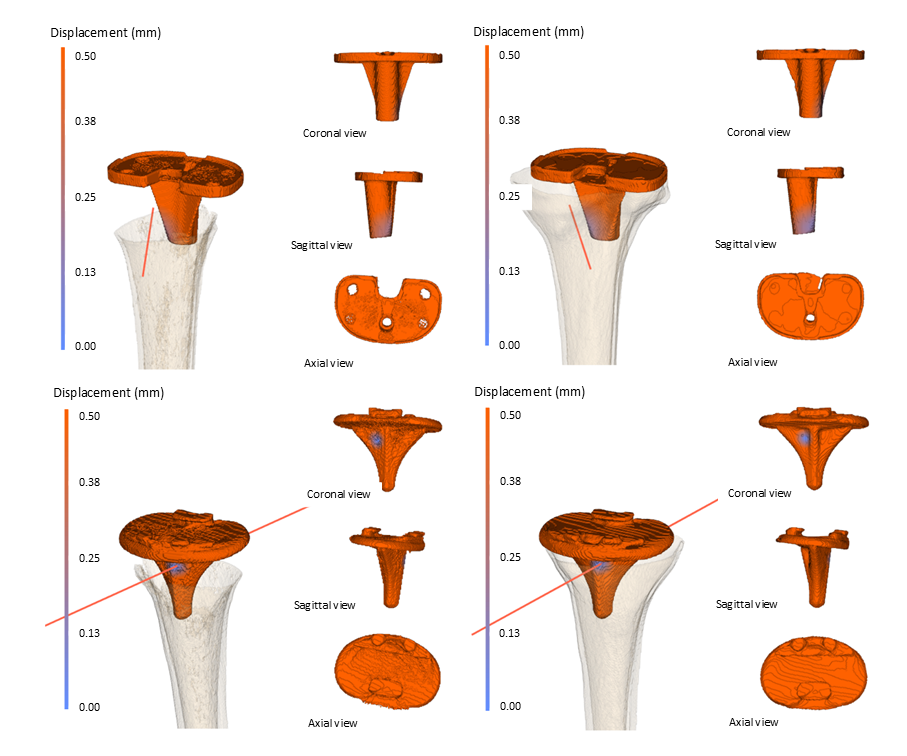
(c) Two symptomatic samples of the real-world patient evaluation dataset (PE).


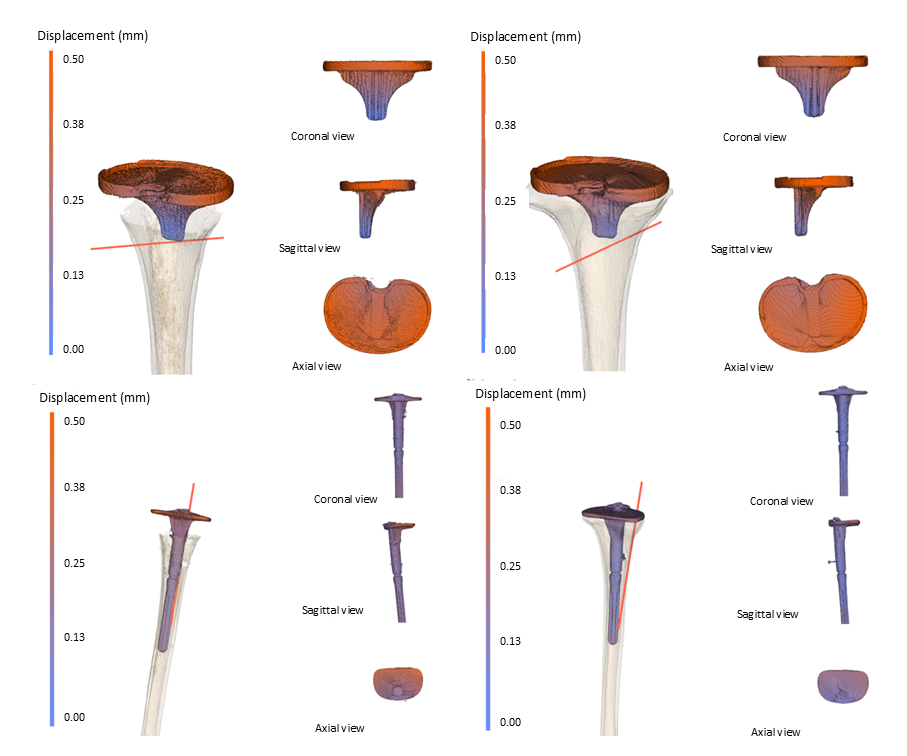
 (d) Two asymptomatic samples of the real-world patient evaluation dataset (PE).

**Fig. 6**: Visualization of displacement for current (first column) and proposed (second column) approach for an example of a cadaveric sample (a), a reproducibility sample (b), and a patient evaluation sample (c+d). 3D model of the cortical bone, the rotation axis and the tibial implant component with a blue-to-red heatmap encoding the displacement measurement are shown.


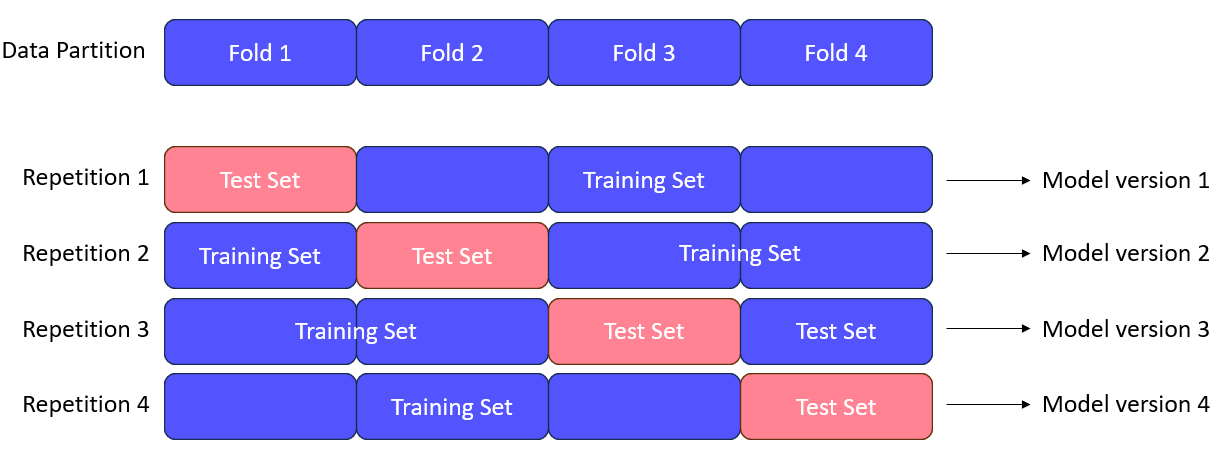


**Fig. 7**: Illustration of 4-fold cross-validation, which is executed for each model configuration.

# C. Model Selection

For the final evaluation on the patient evaluation dataset (PE), one out of six models was selected to test the best model on unseen, real-world data. The model selection was based on multiple criteria: First, we identified the best performers considering segmentation performance (Table 1) and methodological error (Table 3). Considering both segmentation metrics, DSC and HD95, and the median error of all three displacement measurements, the models Full 3D (97.47% DSC, 0.55mm HD95, 0.075 ∆ rScrew (mm), 0.065 ∆ MTPM (mm), 0.035 ∆ mTRE (mm)) and Cortex 3D (95.56% DSC, 0.58mm HD95, 0.085 ∆ rScrew (mm), 0.060 ∆ MTPM (mm), 0.035 ∆ mTRE (mm)) were the best performers. As both models performed very similarly with no clear superiority of one model across all metrics, we based our final model selection on applicability in the downstream task of registration. In the registration process, the correlation coefficient quantified how well the gray-level intensities around the mesh object agree with the target image and it served as metric in the registration optimization method [16]. In our case, a low correlation coefficient meant that the varus CT scan may be misaligned with the varus CT scan, which needed to be confirmed by visual inspection of the registered segmentation masks (Fig. 8). A registration failure was defined as a clear visual misalignment and correlation coefficient close to zero for at least one of the components, which required manual correction by the operator (Fig. 8). The model Full 3D had two registration failure cases, whereas the model Cortex 3D model had no failures.

In the end, the model Cortex 3D was chosen as the final model because it had no registration failures which benefits the aim for increased automation and the tibia segmentation protocol follows closer the protocol of the current approach [8]. The segmentation of the tibia cortex allowed the registration process to choose from more points (i.e., inside and outside of cortex) and the tibia cortex has a more distinct shape, which is expected to make the registration more stable. We believe this to be the reason for the absent of failure cases for model Cortex 3D.

| Method | ∆ rScrew (deg) ↓ | ∆ MTPM (mm) ↓ | ∆ mTRE (mm) ↓ |
| --- | --- | --- | --- |
| **Current** | **0.075 (0.058–0.112)** | **0.080 (0.057–0.099)** | **0.050 (0.031–0.064)** |
| Cortex 2D | 0.080 (0.063–0.123) | 0.070 (0.06–0.092) | 0.040 (0.034–0.056) |
| **Cortex 3D (Final)** | **0.085 (0.067–0.101)** | **0.060 (0.055–0.088)** | **0.035 (0.028–0.053)** |
| Full 2D | 0.090 (0.068–0.122) | 0.070 (0.061–0.094) | 0.040 (0.034–0.055) |
| Full 3D | 0.075 (0.059–0.093) | 0.065 (0.055–0.082) | 0.035 (0.031–0.051) |
| Cortex+Inside 2D | 0.090 (0.066–0.109) | 0.065 (0.06–0.091) | 0.030 (0.029–0.053) |
| Cortex+Inside 3D | 0.075 (0.059–0.108) | 0.065 (0.052–0.088) | 0.040 (0.033–0.054) |
| p-value  Current vs. Cortex 3D | 1 | 0.3 | 0.07 |

Table 3: Comparison methodological error for current and proposed approach using different segmentation models: Median error (∆) and 95% CI for all three displacement parameters (i.e., rScrew, MTPM, mTRE) and the p-value of pairwise comparison between current and the proposed approach with the final model (Cortex 3D). The arrow ↓ indicates that low values are preferable.


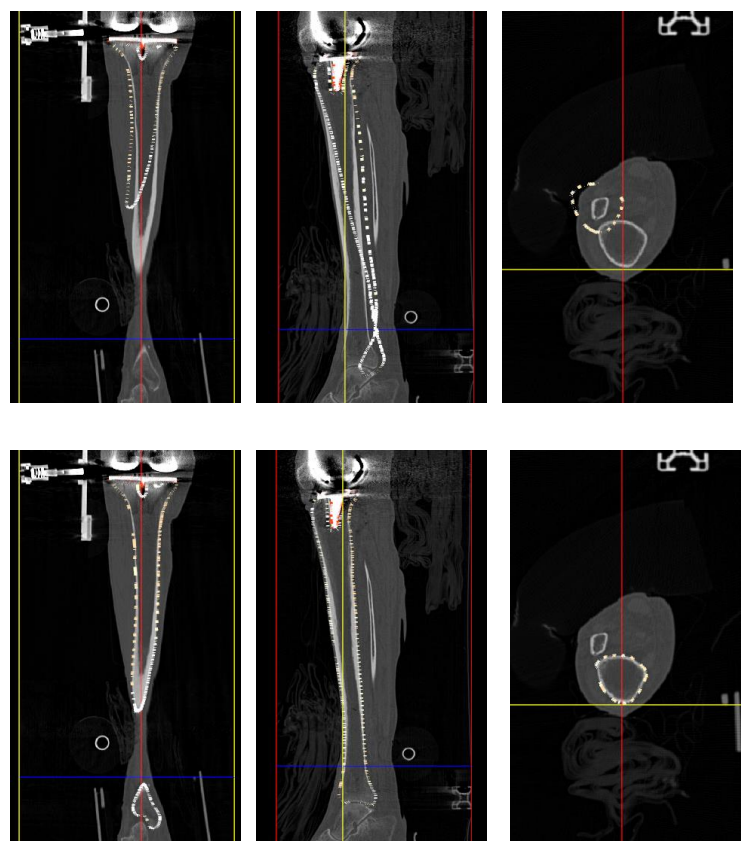


**Fig. 8**: Example of registration failure for tibia bone segmentation masks from model Full 3D: The CT scans are shown in coronal (first column), sagittal (second column), axial (third column) with overlaying polygon outlines of the implant (red) and the tibia bone (white). The first row shows a misalignment of the tibia bone with the underlying CT scan after the registration step, which is also indicated by a correlation coefficient of 0.32. The second row shows the registration result after user intervention (i.e., manual correct of the alignment before the registration step), which improves the correlation coefficient to 0.89.

# D Qualitative Segmentation Examples

Fig. 9 shows examples of segmentation masks generated with the region-growing algorithm, the reference mask for nnUNet training and the predicted mask of the trained 3D nnUNet.


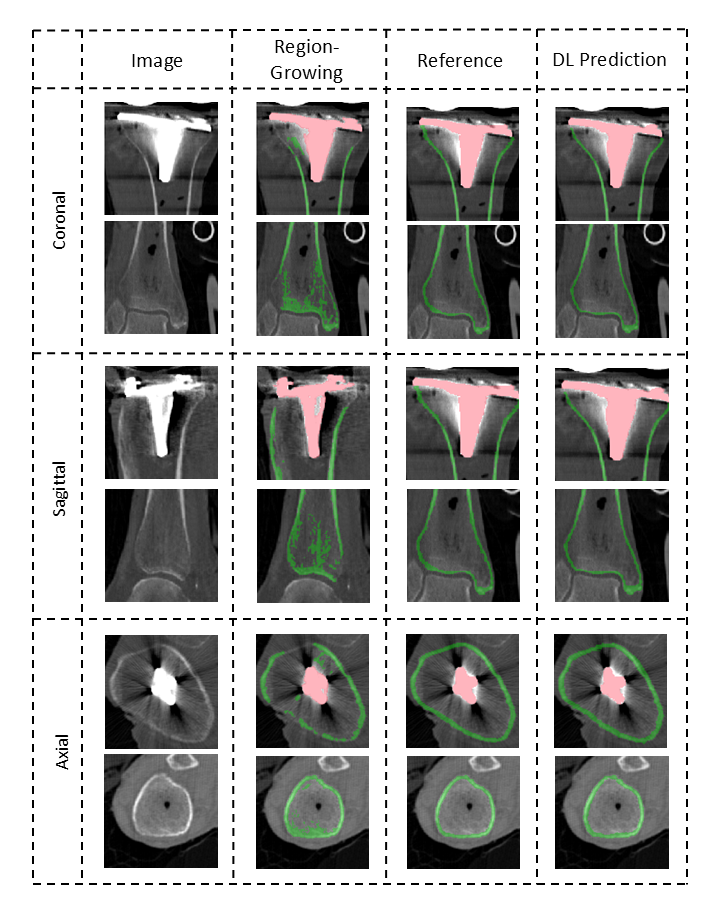


**Fig. 9**: Examples of segmentation masks in all three planes, i.e., coronal, sagittal and axial: Mask generated with region-growing algorithm and used by the current approach (second column); Reference mask used for nnUNet training and evaluation (third column); Prediction by the trained 3D nnUNet (fourth column).

# E. Patient Data Results - descriptive statistics and p-values

In this section, additional descriptive statistics of patient data results (Table 4) and p-values for pairwise comparison of results between different operators and patient groups (Table 5, 6) are reported. Bland-Altmann plots for comparison between current and proposed approach are shown in Fig. 10.

| **Measurements** | | **O1** | **O2** | **O3** | **DL** |
| --- | --- | --- | --- | --- | --- |
| **rScrew (deg)** | **L** | 0.82 (0.93–1.71) | 0.84 (0.92–1.74) | 0.87 (0.92–1.66) | 0.77 (0.89–1.63) |
|  | **F** | 0.39 (0.30–0.57) | 0.40 (0.31–0.60) | 0.40 (0.30–0.61) | 0.40 (0.31–0.62) |
|  | **A** | 0.52 (0.49–0.65) | 0.49 (0.45–0.58) | 0.51 (0.47–0.59) | 0.44 (0.40–0.50) |
| **MTPM (mm)** | **L** | 1.02 (1.05–1.77) | 1.03 (1.05–1.63) | 1.04 (1.01–1.50) | 0.90 (0.95–1.45) |
|  | **F** | 0.64 (0.49–0.81) | 0.64 (0.50–0.84) | 0.64 (0.48–0.84) | 0.64 (0.47–0.78) |
|  | **A** | 0.64 (0.59–0.76) | 0.62 (0.57–0.70) | 0.62 (0.57–0.71) | 0.54 (0.48–0.60) |
| **mTRE (mm)** | **L** | 0.55 (0.58–1.02) | 0.58 (0.59–0.85) | 0.56 (0.56–0.78) | 0.53 (0.52–0.74) |
|  | **F** | 0.40 (0.33–0.59) | 0.41 (0.33–0.60) | 0.41 (0.32–0.59) | 0.38 (0.29–0.53) |
|  | **A** | 0.40 (0.37–0.47) | 0.39 (0.36–0.45) | 0.37 (0.36–0.45) | 0.33 (0.30–0.39) |

Table 4: Median and 95% CI of displacement outcome parameters for loose, fixed and asymptomatic patient data (P), for all three displacement outcome parameters and all four operators.

| **Operators** | **rScrew (deg)** | | **MTPM (mm)** | | **mTRE (mm)** | |
| --- | --- | --- | --- | --- | --- | --- |
|  | **p-value**  **L – F** | **p-value**  **L -A** | **p-value**  **L – F** | **p-value**  **L -A** | **p-value**  **L – F** | **p-value**  **L -A** |
| **O1** | 0.007(**) | < 0.0001(**) | 0.002(*) | < 0.0001(**) | 0.015(*) | < 0.0001(**) |
| **O2** | 0.002(**) | < 0.0001(**) | 0.003(*) | < 0.0001(**) | 0.019(*) | < 0.0001(**) |
| **O3** | 0.001(**) | < 0.0001(**) | 0.003(*) | < 0.0001(**) | 0.022(*) | < 0.0001(**) |
| **DL** | 0.003(*) | < 0.0001(**) | 0.003(*) | < 0.0001(**) | 0.039(*) | < 0.0001(**) |

Table 5: P-values of pairwise comparison between loose and fixed (L – F) or loose and asymptomatic (L – A) subgroups for the current approach by three huma operators (i.e., O1, O2, O3) and the proposed approach (DL).

| **Combination** | **rScrew(deg)** | **MTPM(mm)** | **mTRE(mm)** | |
| --- | --- | --- | --- | --- |
| **O1 – DL** | < 0.001 | < 0.001 |  | < 0.001 |
| **O2 – DL** | < 0.001 | < 0.001 |  | < 0.001 |
| **O3 – DL** | < 0.001 | < 0.001 |  | < 0.001 |
| **O1 – O2** | 0.25 | 0.83 |  | 0.871 |
| **O1 – O3** | 0.15 | 0.40 |  | 0.06 |
| **O2 – O3** | 0.66 | 0.34 |  | 0.13 |

Table 6: P-values for pairwise comparison of results from all four operators, i.e., current approach by human operators (O1, O2, O3) and the proposed approach (DL).


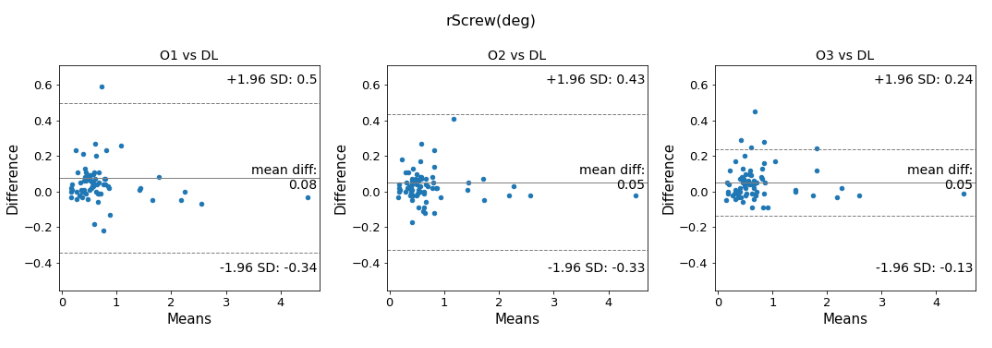

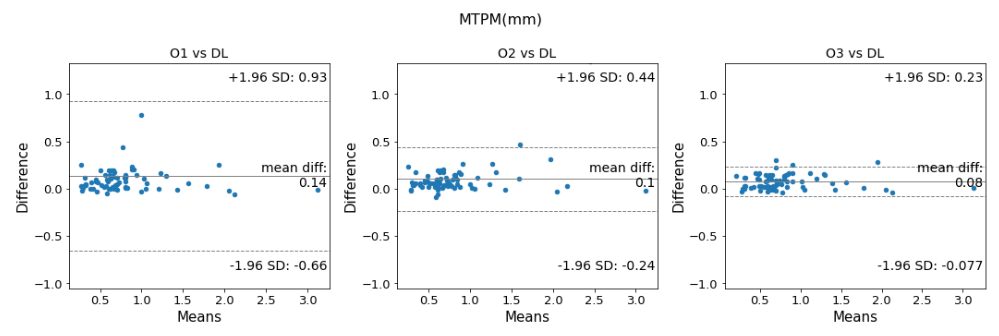

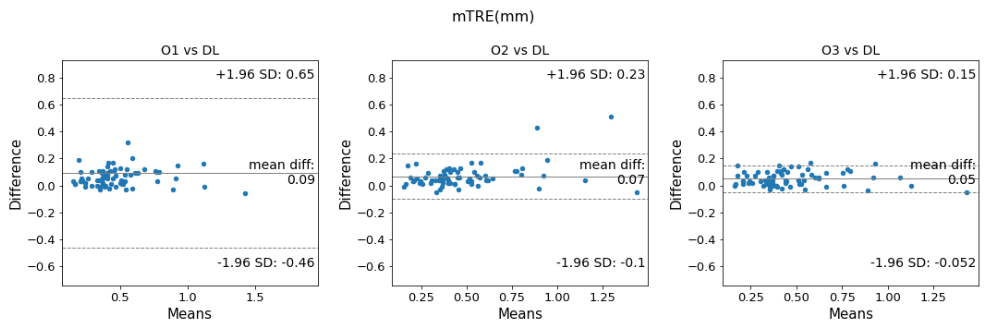


Fig. 10: Bland-Altman plots for the comparison between the current (O1, O2, O3) and proposed (DL) approach for all three displacement measurements (rScrew(deg) – first row. MTPM(mm) – second row, mTRE(mm) – third row).

# References

24. Ronneberger O, Fischer P, Brox T (2015) U-Net: Convolutional networks for biomedical image segmentation. In: *International Conference on Medical Image Computing and Computer-Assisted Intervention (MICCAI)*, 234–241. Springer. https://doi.org/10.1007/978-3-319-24574-4_28

25. Paszke A, Gross S, Massa F, Lerer A, Bradbury J, Chanan G, Killeen T, Lin Z, Gimelshein N, Antiga L, et al. (2019) PyTorch: An imperative style, high-performance deep learning library. In: *Advances in Neural Information Processing Systems (NeurIPS)*, 8024–8035. https://doi.org/10.48550/arXiv.1912.01703

26. Chen LC, Papandreou G, Kokkinos I, Murphy K, Yuille A (2017) DeepLab: Semantic image segmentation with deep convolutional nets, atrous convolution, and fully connected CRFs. IEEE Trans. Pattern Anal. Mach. Intell. 40: 834–848. https://doi.org/10.1109/TPAMI.2017.2699184

27. Drozdzal M, Vorontsov E, Chartrand G, Kadoury S, Pal C (2016) The importance of skip connections in biomedical image segmentation. In: Deep Learning and Data Labeling for Medical Applications (Carneiro G, et al., eds.), 179–187. Springer.
